# Genetic characterization and clonal analysis of carbapenemase-producing *Escherichia coli* and *Klebsiella pneumoniae* from canine and human origins

**DOI:** 10.3389/fvets.2024.1464934

**Published:** 2024-11-25

**Authors:** Nwai Oo Khine, Asad Ali Shah, Tanittha Chatsuwan, Jitrapa Yindee, Natthapong Supimon, Imporn Saenkankam, David John Hampson, Nuvee Prapasarakul

**Affiliations:** ^1^Department of Microbiology, Faculty of Veterinary Science, Chulalongkorn University, Bangkok, Thailand; ^2^Department of Microbiology, Faculty of Medicine, Chulalongkorn University, Bangkok, Thailand; ^3^School of Veterinary Medicine, Murdoch University, Perth, WA, Australia; ^4^Center of Excellency in Diagnosis and Monitoring of Animal Pathogens (CEDMAP), Bangkok, Thailand

**Keywords:** carbapenemase-producing Enterobacteriaceae, clonal relatedness, *Escherichia coli*, genetic characterization, *Klebsiella pneumoniae*

## Abstract

**Introduction:**

Carbapenem-resistant Enterobacterales (CRE), particularly carbapenemase-producing *Escherichia coli and Klebsiella pneumoniae*, pose a significant global health challenge due to their resistance to last-resort antibiotics. This study investigates the genetic characteristics and clonal relationships of CRE isolated from canine and human clinical samples in Bangkok to understand potential interspecies transmission.

**Methods:**

Fifty-two CRE isolates were collected from 477 clinical samples from dogs and humans at Chulalongkorn University between 2017–2021. Bacterial species were identified using MALDI-TOF, and antimicrobial resistance was confirmed through broth microdilution testing. Genetic analyses included plasmid replicon typing, multilocus sequence typing (MLST), whole genome sequencing (WGS), and pulsed-field gel electrophoresis (PFGE) to assess resistance genes and clonal relatedness.

**Results:**

CRE isolates from both species exhibited genetic variability with high ARG counts, particularly in human isolates. MLST identified ST410 in most E. coli isolates from both dogs and humans, and IncFIA/IncFIB plasmids were predominant among blaNDM-positive isolates. PFGE patterns and SNP analysis showed no clonal relationship between canine and human isolates, suggesting independent acquisition pathways for CRE in the two hosts.

**Discussion:**

The study highlights the absence of direct clonal transmission between canine and human isolates but reveals overlapping sequence types and plasmid types. The findings underscore the potential for interspecies transmission under certain conditions, emphasizing the importance of a One Health approach for monitoring CRE in both human and animal populations.

## 1 Introduction

The increase in infections caused by antimicrobial resistant bacteria has become a critical global health problem, leading to increased morbidity and mortality rates (1). Among the most concerning multidrug-resistant pathogens are those resistant to last-resort antibiotics such as colistin, carbapenems, and extended-spectrum beta-lactamase-producing (ESBL) bacteria (2). Carbapenems, which are broad-spectrum beta (*β*)-lactam antibiotics, are commonly used in the treatment of life-threatening Enterobacterales infections. Unfortunately, the growing prevalence of carbapenem-resistant Enterobacterales (CRE) poses a significant challenge in terms of treatment options (3). The acquisition of carbapenem-hydrolyzing enzymes, known as carbapenemases, has led to the development of carbapenem resistance in these pathogens (3). Carbapenemases belong to different enzyme families, with the most notorious being *KPC, NDM, OXA-48, IMP*, and *VIM,* and their encoding genes are often located on mobile genetic elements such as plasmids (4).

The emergence of CRE in companion animals has raised concerns about the potential role of animals in disseminating CRE to humans and the environment, whether through clonal spread or plasmid transmission (5). Strict control of CRE infections in human and animal healthcare settings is challenging due to the ability of these organisms to colonize the gastrointestinal tract and go undetected. Therefore, comprehensive monitoring and characterization of carbapenem-resistant isolates from companion animals are necessary to assess their potential for human transmission.

While CRE infections in humans are commonly reported, there have been relatively few reports of CRE from companion animals, particularly in Thailand. However, the co-selection or co-localization of resistance genes on the same plasmids suggests a potential route of transmission (6). Given the close contact and shared environment between humans and companion animals, the role of these animals in the potential transmission of CRE to humans is a matter of public health concern (7). Therefore, it is essential to explore the acquisition of carbapenem resistance in CRE isolates from companion animals such as dogs to seek genetic evidence for intraspecies transmission. The purpose of this study was to characterize carbapenemase-producing Enterobacterales in dogs and humans, with an emphasis on identifying shared plasmid types, resistance genes, and clonal groups to help understand the potential for cross-species spread.

## 2 Materials and methods

### 2.1 Background and source of carbapenem-resistant isolates

A total of 52 carbapenem-resistant isolates were recovered from 477 clinical samples submitted for routine diagnostic workup to the Chulalongkorn University Faculty of Veterinary Science, Veterinary Diagnostic Unit between 2017–2021 (Supplementary Tables S1, S2). Samples were plated to MacConkey agar, and lactose positive isolates were identified as *Klebsiella pneumoniae* or *Escherichia coli* using IMViC biochemical tests and MALDI-TOF MS (Bruker-Daltonics, Bremen, Germany) (8). Initial screening of the antimicrobial susceptibilities of the isolates was undertaken using the Vitek 2® (bioMérieux, France). Fifty-two carbapenem-resistant isolates were obtained (from 477 samples, 10.9%), with most originating from urinary tract infections (UTIs). Ten CR isolates from dogs, comprising *K. pneumoniae* (*n* = 3) and *E. coli* (*n* = 7) were chosen for further examination. Additionally, 18 human clinical isolates, comprising *K. pneumoniae* (*n* = 10) and *E. coli* (*n* = 8) obtained from patients with UTIs attending the Faculty of Medicine, Chulalongkorn University in the same period were included in this investigation. The minimum inhibitory concentrations (MIC) against relevant antibiotics for all probable human CRE isolates previously had been determined, and all showed resistance to cefepime, cefotaxime, cefoxitin, and ceftazidime. The bacterial strains used in this study were approved for use by Chulalongkorn University’s Institutional Biosafety Committee under Biosafety Use Protocol number 203104 and a Certificate of Expedited Review Approval from the Institutional Review Board of the Faculty of Medicine. Chulalongkorn University also complies with the International Conference on Harmonization in Good Clinical Practice (ICH-GCP), IRB No. 0283/65.

### 2.2 Antimicrobial resistance phenotypes

All the bacterial isolates from humans and dogs underwent broth-micro dilution antimicrobial susceptibility testing utilizing EUVSEC2 Sensititre plates. CLSI clinical breakpoints were used for interpreting the MIC results. Cefoxitin, ertapenem, imipenem, meropenem, ceftazidime, cefepime, cefotaxime, and temocillin were all included on the plates. Carbapenem resistance was defined as a MIC of 2 μg/mL for ertapenem or a MIC of 4 μg/mL for imipenem or meropenem (9). Metallo-beta-lactamase (MBL) production was confirmed using the double disc potentiation test (10). Briefly, the tested bacteria were inoculated as a lawn on Mueller-Hinton agar plates and two imipenem disks (10 μg) and two meropenem disks (10 μg) were placed on the plates. A 5 mL volume of 0.5 M EDTA solution was added to one imipenem and one meropenem disk. MBL production was indicated by an increase in the zone size ( ≥ 5 mm) around the EDTA-containing disks due to inhibition of the carbapenemase enzyme in the presence of the metal-chelating agent EDTA.

### 2.3 Detection of acquired carbapenemase genes

Bacterial DNA was extracted from all 28 CRE isolates using the Thermo Scientific GeneJET Genomic DNA Purification Kit (Thermo Fisher Scientific). Multiplex-PCR for 11 acquired carbapenemase genes (*bla*_IMP_, *bla*_VIM_, *bla*_SPM_, *bla*_GIM_, *bla*_SIM_, *bla*_KPC_, *bla*_NDM_, *bla*_AIM_, *bla*_DIM_, *bla*_BIC_, and *bla*_OXA-48_) was applied following a previously published protocol (11). Positive products were subjected to Sanger sequencing for confirmation of the presence of the genes.

### 2.4 Plasmid replicon typing

A set of multiplex and simplex PCRs was used to analyze the Enterobacterales plasmid replicon types. The PCR settings and primers for 18 different plasmid replicon types were carried out in accordance with a previously described technique (12). In a brief, PCR amplification was carried out at 94°C for 5 min, then 30 cycles at 94°C for 1 min, 60°C for 30 s, and 72°C for 1 min, with the final extension of 1 cycle at 72°C for 5 min. An exception to this was the replicon type F-simplex PCR where annealing was at 52°C (12).

### 2.5 Pulsed-field gel electrophoresis

The clonal relatedness between the canine and human isolates of *E. coli* (*n* = 15) and *K. pneumoniae* (*n* = 13) that showed resistance to carbapenem was investigated using pulsed-field gel electrophoresis (PFGE), following the standard protocol of the Centers for Disease Control and Prevention (13). The genomic DNA was digested with restriction enzyme *XbaI* (Thermo Scientific). The Bio-Rad CHEF-DRIII system was used for gel electrophoresis, with a 200 V field at an angle of 120° and run for 17–20 h, incorporating *Salmonella* serovar *Braenderup* H9812 (*n* = 1) DNA as a standard. Dendrograms were visualized by using the GeneTool program (Syngene, India) and analyzed with the GeneDirectory program (Syngene, India).

### 2.6 Multilocus sequence typing (MLST)

The non-clonally related *E. coli* and *K. pneumoniae* isolates identified using PFGE were further analyzed by MLST to define their sequence types (STs). For *E. coli,* genes encoding seven housekeeping functions including isocitrate/isopropyl malate dehydrogenase (*icd*), ATP/GTP binding motif (*recA*), adenylate kinase (*adk*), DNA gyrase (*gyrB*), malate dehydrogenase (*mdh*), adenyl succinate dehydrogenase (*purA*) and fumarate hydratase (*fumc*) were amplified using the MLST Achtman scheme (14). For *K. pneumoniae*, seven housekeeping genes including those encoding the beta-subunit of RNA polymerase B (*rpoB*), glyceraldehyde 3-phosphate dehydrogenase (*gapA*), malate dehydrogenase (*mdh*), phosphoglucose isomerase (*pgi*), phosphoporine E (*phoE*), translation initiation factor 2 (*infB*), and periplasmic energy transducer (*tonB*) were amplified (15). The MLST databases at: http://mlst.warwick.ac.uk/mlst/dbs/Ecoli and http://pubmlst.org/kpneumoniae were used to determine alleles and STs for members of the two species.

### 2.7 Whole genome sequencing for resistome analysis

DNA extraction of CRE isolates (15 *E. coli* and 6 *K. pneumoniae*) was performed using the ZymoBIOMICS DNA Miniprep Kit according to the manufacturer’s instructions. The extracted DNA samples were checked for quality using NanoDrop spectrophotometry as recommended (A260/280 1.8 ~ 2), and then submitted for sequencing using the Illumina NovaSeq PE150 platform. Adapter sequences and low-quality sequences were removed using Trimmomatic v.0.36.5 (16). Hybrid assembly was performed using Unicycler for accurate genome construction. Core genome SNPs were identified using the Galaxy platform’s SNP site tool^1^ using SPADES (Galaxy Version 3.15.4 + galaxy1), and a phylogenetic SNP tree was constructed in IQTree with maximum likelihood methods. Distance in SNPs was computed to assess clonal relationships among isolates, visualized through the iTOL platform for comparative genomic analysis. The assemblies were analyzed using the pipeline from the Centers for Genomic Epidemiology^2^ (17), including species identification (KmerFinder 2.1), Multilocus Sequence Typing (MLST 1.6), and presence of antimicrobial resistance genes (ResFinder 2.1). Acquired antimicrobial resistance genes (ARGs), also were identified using ABRicate (Galaxy Version 1.0.1).

### 2.8 Data availability

The datasets generated and/or analyzed during the current study are available in the National Library of Medicine repository BioProject: PRJNA1087749.

## 3 Results

### 3.1 Phenotypic and genotypic evidence for carbapenemase resistance

All 15 *E. coli* isolates (*n* = 7 from dogs, *n* = 8 from humans) showed evidence for carbapenemase production in the double disc potentiation test. For the *K. pneumoniae* isolates (*n* = 3 from dogs, *n* = 10 from humans) only 12 out of the 13 were found to be positive for carbapenamase production in the EUVSEC2 Sensititre plates. The KPFM5 isolate, which tested negative for carbapenemase production, was obtained from a human patient. The antimicrobial susceptibility testing results for the CR *E. coli* and *K. pneumoniae* isolates are showing in Table 1. All *E. coli* isolates from dogs displayed resistance to either ertapenam, meropenam or imipenem.

**Table 1:** Results of antimicrobial susceptibility testing for carbapenem resistant *E. coli* and *K. pneumoniae* isolates.

No.		Bacterial isolates	Source	MIC by using Sensititre							
				Cefoxitin	Ertapenem	Imipenem	Meropenem	Ceftazidime	Cefepime	Cefotaxime	Temocillin
				FOX	ETP	IMI	MERO	TAZ	FEP	FOT	TRM
1	*Escherichia coli*	EC00	Dog	>64	>2	4	>16	>128	>32	>64	128
2		EC01	Dog	>64	>2	4	>16	>128	>32	>64	>128
3		EC02	Dog	>64	>2	4	>16	>128	>32	>64	>128
4		EC03	Dog	>64	>2	4	>16	>128	>32	>64	>128
5		EC05	Dog	>64	>2	4	>16	>128	>32	>64	>128
6		EC06	Dog	>64	>2	4	>16	>128	>32	>64	>128
7		EC09	Dog	>64	>2	4	>16	>128	>32	>64	128
8		ECFM03	Human	>64	>2	>16	>16	>128	>32	>64	>128
9		ECFM04	Human	>64	>2	8	>16	>128	>32	>64	>128
10		ECFM05	Human	R	R	R	R	R	R	R	S
11		ECFM06	Human	R	R	R	R	R	R	R	S
12		ECFM07	Human	>64	>2	8	4	>128	>32	>64	>128
13		ECFM08	Human	>64	>2	8	4	>128	>32	>64	>128
14		ECFM09	Human	R	R	R	R	R	R	R	R
15		ECFM10	Human	>64	>2	2	2	>128	>32	>64	>128
16	*Klebsiella pneumoniae*	KP01	Dog	>64	>2	>16	>16	64	>32	>64	>128
17		KP02	Dog	>64	>2	2	1,S	>128	8,I	>64	>128
18		KP03	Dog	>64	>2	>16	>16	>128	>32	>64	>128
19		KPFM 1	Human	>64	>2	>16	>16	>128	>32	>64	>128
20		KPFM 2	Human	>64	>2	4	8	64	>32	>64	>128
21		KPFM 3	Human	>64	>2	>16	>16	>128	>32	>64	>128
22		KPFM 4	Human	8	>2	0.25	0.5	0.5	0.25	0.5	>128
23		KPFM 5	Human	2	≤0.015	≤0.12	≤0.03	1	4	8	2
24		KPFM 6	Human	>64	>2	>16	>16	64	>32	>64	>128
25		KPFM 7	Human	I	R	R	R	R	R	R	R
26		KPFM 8	Human	>64	>2	8	>16	>128	>32	>64	>128
27		KPFM 9	Human	R	R	R	R	R	R	R	R
28		KPFM 10	Human	2	>2	>2	>2	4	4	32	>128

All 7 canine CR *E. coli* isolates were positive for the *bla*_NDM_ gene by PCR, while one of the three canine CR *K. pneumoniae* isolates contained *bla*_NDM_ and two were positive for *bla*_OXA_ (Table 2). For the human isolates, all 8 *E. coli* isolates that were positive in phenotypic testing contained both *bla*_NDM_ and *bla*_OXA_ genes, while in the case of *K. pneumoniae*, all 10 isolates contained *bla*_OXA_ and two also were *bla*_NDM_ positive (Table 3).

**Table 2:** Acquired carbapenemase genes and sequence types of canine and human *E. coli* isolates.

No	Source	Bacterial isolate	Carbapenemase gene		Sequence type (ST)	Plasmid typing
			NDM variants	OXA		
1	Dogs	EC00	*NDM-5*		ST410	IncFIA, IncFIB, IncY
2		EC01	*NDM-5*		ST410	IncFIA and IncFIB,
3		EC02	*NDM-5*		ST410	IncFIA and IncFIB
4		EC03	*NDM-5*		ST410	IncFIA and IncFIB
5		EC05	*NDM-5*		ST410	IncFIA and IncFIB
6		EC06	*NDM-5*		ST410	IncFIA and IncFIB
7		EC09	*NDM-5*		ST410	IncFIA, IncY, IncFIB
8	Humans	ECFM03		*OXA-48*	ST410	IncFIA, IncFIB, ColRNAI
9		ECFM04	*NDM-5*	*OXA-48*	ST648	IncFIA and IncFIB
10		ECFM05	*NDM-5*	*OXA-48*	ST410	IncFIA and IncFIB
11		ECFM06	*NDM-5*	*OXA-48*	ST410	IncFIA, IncFIB, ColRNAI
12		ECFM07	*NDM-5*	*OXA-48*	ST410	IncFIA, IncFIB, IncI1
13		ECFM08	*NDM-5*	*OXA-48*	ST410	IncFIA and IncFIB
14		ECFM09	*NDM-5*	*OXA-48*	ST410	IncFIA, IncFIB, IncHI, ColRNAI
15		ECFM10	*NDM-5*	*OXA-48*	ST410	IncFIA and IncFIB

**Table 3:** Acquired carbapenemase genes and sequence types of canine and human *K. pneumoniae* isolates.

No.	Source	Bacterial isolate	Carbapenemase gene		Sequence types	Plasmid Typing
			NDM variants	OXA variants		
1	Dogs	KP01		*OXA-48*	ST16	IncFIA and IncFIB
2		KP02		*OXA-48*	ST147	IncA/C, ColKP3, IncFIB
3		KP03	*NDM-1*	*-*	ST15	IncFIA, IncFIB, IncHI
4	Humans	KP FM 1	*NDM-1*	*OXA-48*	ST16	IncFIA and IncFIB
5		KP FM 2		*OXA-48*	ST231	IncFIA
6		KP FM 3	*NDM-1*	*OXA-48*	ST340	IncFIA, IncFIB, IncN
7		KP FM 4		*OXA-48*	ST1269	IncC, IncN, IncFII, ColKP3
8		KP FM 5		*OXA-48*	ST5	Inc I1
9		KP FM 6		*OXA-48*	ST231	IncFIA
10		KP FM 7		*OXA-48*	NR	IncFIA
11		KP FM 8		*OXA-48*	ST231	IncFIA
12		KP FM 9		*OXA-48*	NR	IncFIA
13		KP FM 10		*OXA-48*	ST35	Inc I1

NR, non-registered ST type.

### 3.2 Resistome analysis

**Figure 1:**
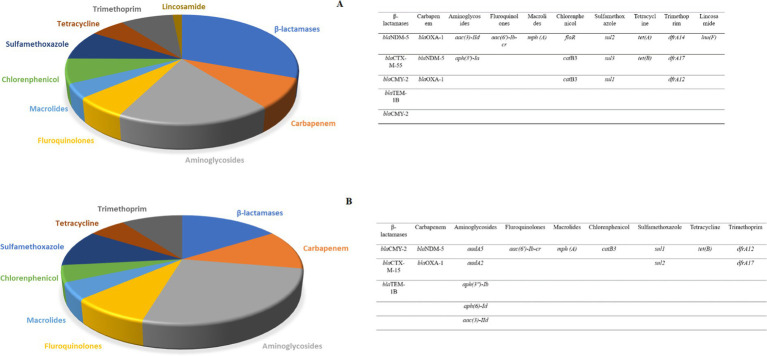
**(A)** The relative number of antibiotic resistance genes found in eight human carbapenem resistant *E. coli* subjected to WGS. The highest abundance was observed for the beta-lactamase group. **(B)** The number of antibiotic resistance genes found in seven canine carbapenem resistant *E. coli*. The highest abundance was observed for the aminoglycoside antibiotics group.

WGS of the 15 CR *E. coli* isolates identified an average of 24 antibiotic-resistance genes (ARGs) in the eight human isolates, while an average of 18 were detected in the seven canine isolates. These ARGs were directed against ten antibiotic groups including beta-lactams, carbapenem, aminoglycosides, fluroquinolones, macrolides, chloramphenicol, sulfamethoxazole, tetracycline, trimethoprim and lincosamide. Notably, ARGs against Lincosamide were exclusively present in human isolates. Among the human isolates, the most prevalent ARGs were from the beta-lactamase group, whereas in the canine isolates, aminoglycoside-resistant genes were most abundant (Figure 1).

**Figure 2:**
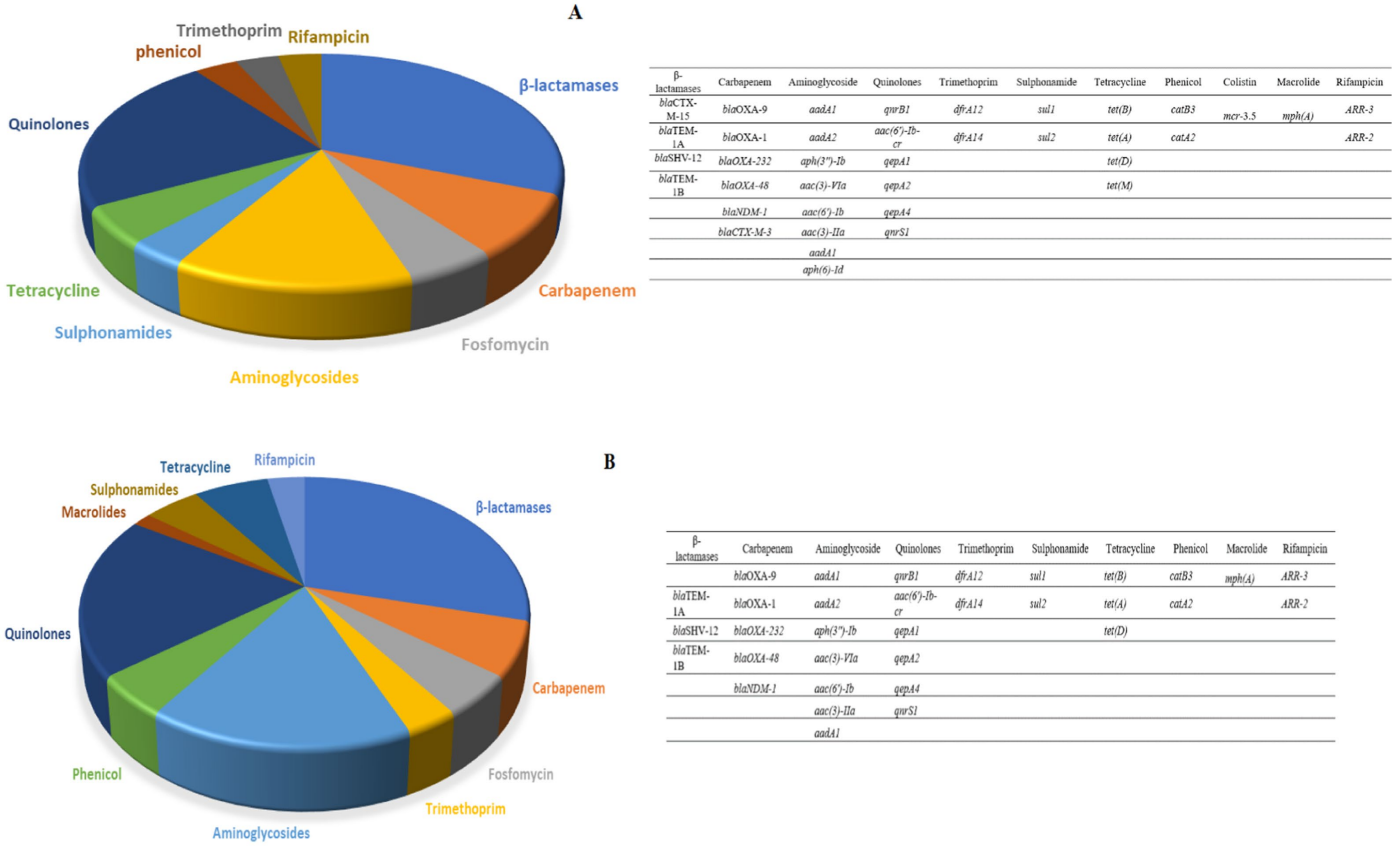
**(A)** The relative number of antibiotic resistance genes found in three human carbapenem resistant *K. pneumoniae* isolates subjected to WGS. **(B)** The number of antibiotic resistance genes found in three canine carbapenem resistant *K. pneumoniae*.

In the case of the six CR *K. pneumoniae* (CRK) isolates that were sequenced, an average of six ARGs directed against beta-lactams, carbapenem, fosfomycin, trimethoprim, aminoglycosides, phenicols, quinolones, macrolides, sulphonamides, tetracycline and rifampicinin were detected in both the canine and human isolates. The *β*-lactams and Quinolones groups displayed the highest ARG abundances (Figure 2). A similar number of antibiotic resistance genes was found in CRK isolates from dogs and humans.

### 3.3 Plasmid replicon typing

PCR based plasmid-replicon typing identified IncFIA, IncFIB, IncA/C, IncY, IncN, and Inc. I1 in the *E. coli* and *K. pneumoniae* isolates. Of these, IncFIA and IncFIB were the most common types among the *bla*_NDM_ positive *E. coli* isolates (Table 2), whereas IncFIA was the most prevalent in the *bla*_OXA-48_ and *bla*_NDM_ positive *K. pneumoniae* isolates (Table 3). A strong association was observed between the PCR based plasmid replicon typing and plasmid finder results using the WGS. Moreover, PCR analysis for Integron types revealed that a class 1 integron was found in all *E. coli* and *K. pneumoniae* isolates.

### 3.4 Pulsed field gel electrophoresis (PFGE)

**Figure 3:**
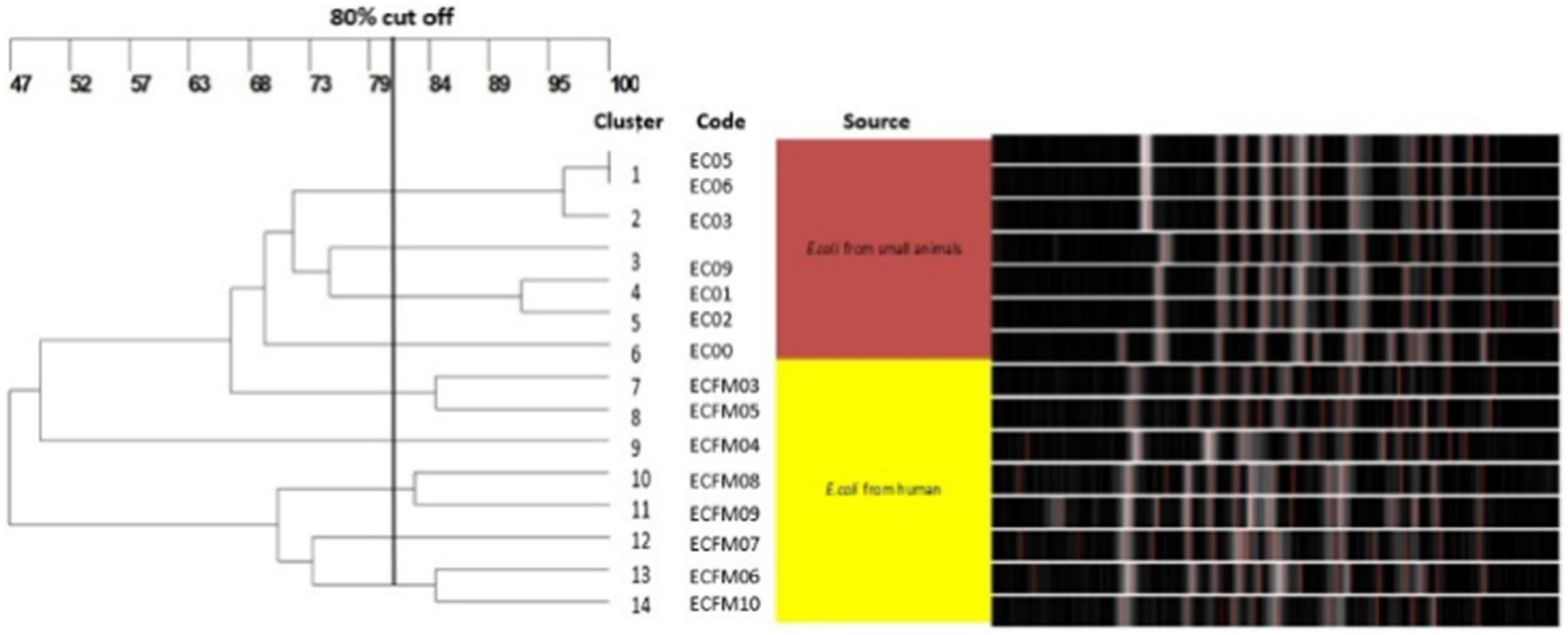
Pulsed-field gel electrophoresis dendrogram showing relationships of carbapenem resistant *E. coli* isolates from dogs and humans.

**Figure 4:**
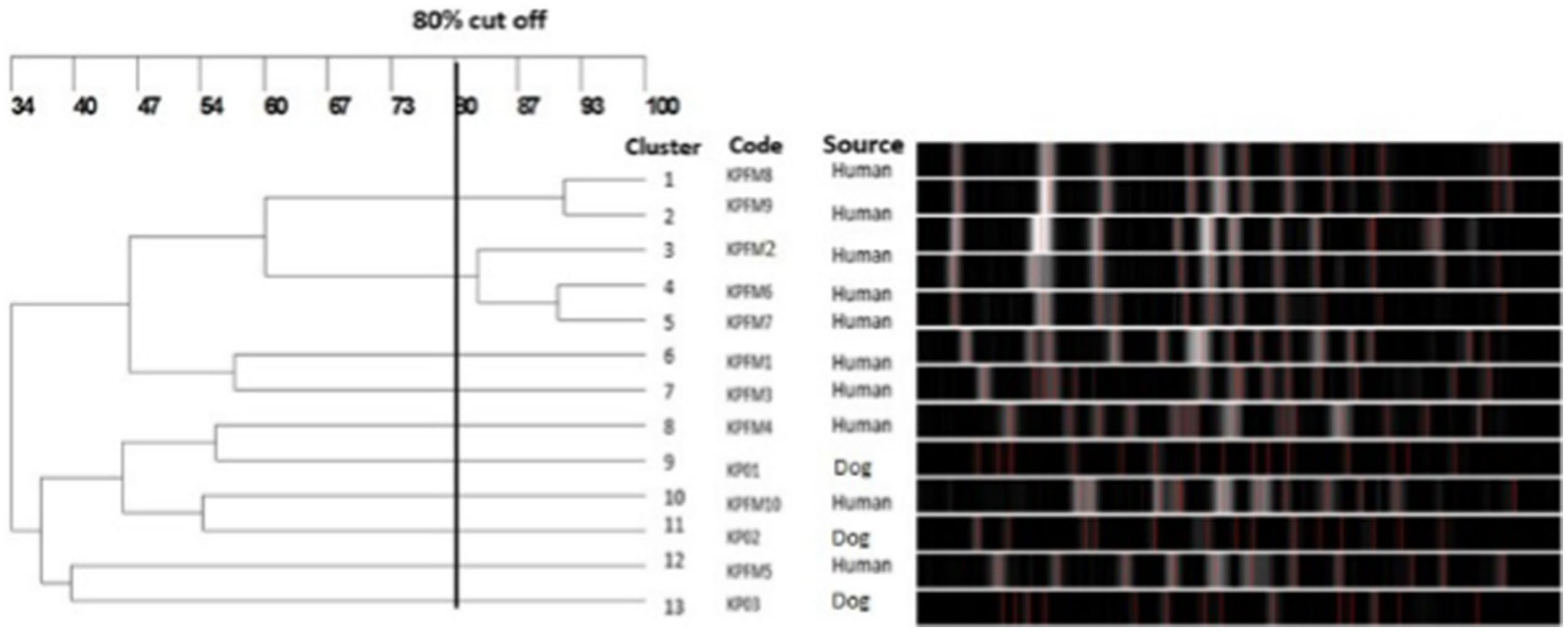
Pulsed-field gel electrophoresis dendrogram showing relationships of carbapenem resistant *K. pneumoniae* isolates from dogs and humans.

To investigate the clonal relatedness of carbapenem resistant *E. coli* and *K. pneumoniae* isolates from dogs and humans, PFGE was performed on all *bla*_NDM_ and *bla*_OXA_ positive isolates. Fourteen PFGE patterns were obtained for the 15 CR *E. coli* isolates. The canine and human isolates did not share any pulsotypes or clonal relationships (Figure 3). Similarly, no shared pulsotypes were detected among the CR *K. pneumoniae* isolates from dogs and humans. High similarity pulsotypes (>80%) were only found between human strains or between canine strains (Figure 4).

### 3.5 Multilocus sequence typing (MLST)

MLST revealed that the CR *E. coli* isolates from humans and dogs belonged to ST 410, except for isolate ECFM04 from a human which was assigned to ST 648 (Table 2). The three *K. pneumoniae* isolates from dogs belonged ST16, ST147 and ST15 respectively, which were not shared with human isolates (Table 3). Two of the 10 human isolates had unregistered STs, three shared ST231, and the other five all belonged to different STs.

### 3.6 SNP analysis

**Figure 5:**
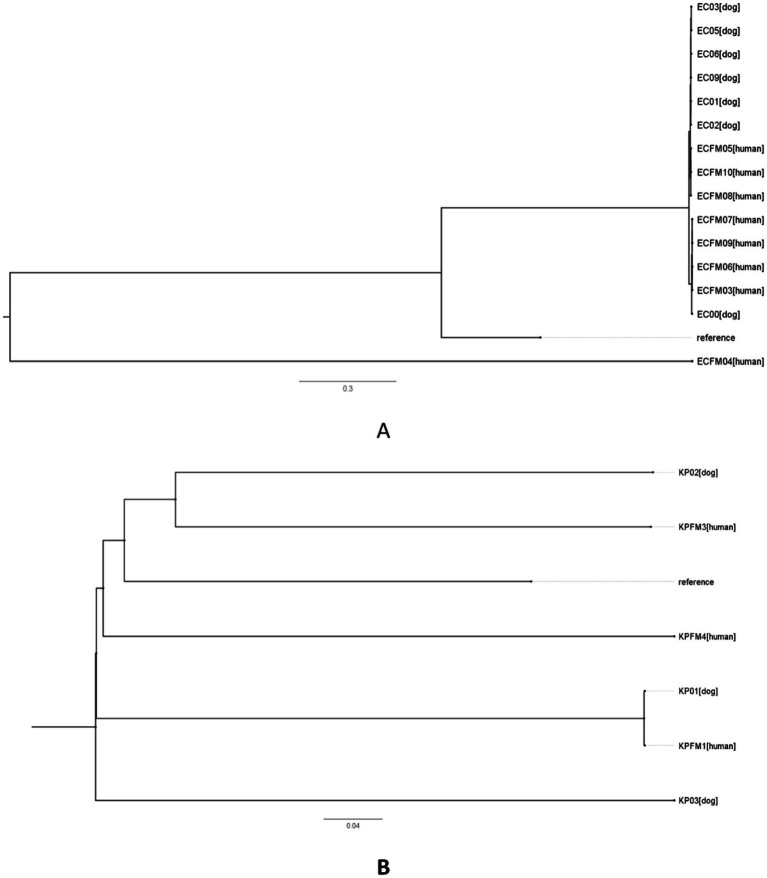
Phylogenetic single nucleotide polymorphisms (SNPs) in carbapenem-resistant *Escherichia coli* (A) and *Klebsiella pneumoniae* (B) strains derived from human and canine sources. The phylogenetic trees based on SNPs were created utilizing core genome SNPs to evaluate the clonal connections among the isolates. Each branch signifies a distinct isolate, with distances reflecting the level of genetic variation in SNPs. The reference strain *E. coli* K12 (accession no. NC_000913.2) was included in A, while *K. pneumoniae* sequence MGH78578 (accession no. NC_009653) was included in B.

Genome sequences were aligned with *K. pneumoniae* sequence MGH78578 and *E. coli* K12 as references for genomic comparisons and phylogenetic tree analysis. The data indicated a greater number of SNP differences, reflecting increased genetic divergence across strains (30–48,026 for *E. coli* and 54–21,067 for *K. pneumoniae*) as illustrated in Figures 5A,B and Supplementary Tables S3, S4.

## 4 Discussion

This study aimed to investigate the genetic characteristics and clonal relatedness of CRE isolates, with a particular focus on the potential inter-host sharing between humans and dogs. The isolates that were studied were obtained from dogs and humans with no known connections, other than their origin in Bangkok from patients with UTIs. This reduced the likelihood of any direct transmission of strains between these individuals. A significant portion of the *E. coli* and *K. pneumoniae* isolates that were examined from both dogs and humans exhibiting phenotypic resistance to carbapenems, and the detection of acquired carbapenemase genes, particularly *bla*_NDM_ and *bla*_OXA_, confirmed the genetic basis for carbapenem resistance. The identification of specific carbapenemase genes, including *bla*_NDM-1_, *bla*_NDM-5_, and *bla_OXA-48_,* further highlighted the diverse mechanisms contributing to carbapenem resistance in these isolates. In the past, a few isolates from human UTIs that shared some genetic characteristics with NDM-5-producing *E. coli* from dogs were reported in Finland and Egypt (18, 19). *E. coli* and *K. pneumoniae*, which produce class D oxacillinases (*OXA-48*), also have been commonly found in dogs in Germany and in humans in West and Central Africa (20–22). In addition to the isolation of NDM-1-producing *E. coli*, the presence of *bla*_OXA-48_ in *E. coli* and *K. pneumoniae* isolated from companion animals (cats, dogs or horses) has been noted (23). A recent study reported the presence of *bla*_KPC-2_, *bla*_IMP-1_, _*bla*VIM-1_, *bla*_NDM-1_, *bla*_NDM-5_, and *bla_OXA-48_* in carbapenem-resistant *E. coli* and *K. pneumoniae* from both healthy and diseased dogs and cats (22). These findings suggest that dogs can serve as reservoirs and potential sources of carbapenemase-producing bacteria, posing a risk for transmission to humans.

Interestingly, our findings indicate a relatively higher count of ARGs in human CR *E. coli* isolates, especially in the beta-lactamase groups, compared to in canine isolates. Various antimicrobial agents, such as beta-lactams, tetracyclines, aminoglycosides, fluoroquinolones have been used for treating UTIs in dogs (24). The presence of CRE with multidrug-resistant characteristics has made the treatment of UTIs in dogs more challenging. Moreover, quinolones are the first choice for treatment in ESBL-producing enterobacterial infections, and a combination of carbapenems with quinolones are applied in carbapenem non-susceptible *K. pneumoniae* infections (25, 26). The consistent presence of certain ARGs, such as those related to *β*-lactams and quinolones, in both canine and human isolates of CRK was quite concerning. This occurrence not only limits available treatment options but also suggests the potential cross-species transmission or existence of shared environmental reservoirs contributing to the spread of resistance. These findings underscore the importance of comprehensive surveillance and molecular epidemiological studies to elucidate the dynamics of ARG dissemination and identify potential intervention strategies.

Plasmid replicon typing provided insights into the diversity of plasmids carrying carbapenemase genes. The detection of various replicon types, mostly IncFIA and IncFIB, suggests that these are the common replicon types associated with plasmids that carry carbapenem resistance genes (27, 28). These replicon types are commonly found in plasmids carrying carbapenemase genes and are known to facilitate their autonomously horizontal transfer between different bacterial strains and species (29, 30). The finding suggests a potential for inter-species transmission and dissemination of carbapenem resistance. This is of significant concern from a One Health perspective, as it highlights the interconnectedness between companion animal and human health. The transmission of carbapenem resistance genes from dogs to humans, or vice versa, can occur through close contact and shared environments, such as in households, veterinary clinics, or animal production settings (31). The presence of common replicon types such as IncFIA and IncFIB in both animal and human isolates suggests a possible shared reservoir of carbapenem resistance genes and underscores the relevance of a One Health approach in addressing the spread of carbapenem resistance. Understanding the genetic elements involved in the transmission of resistance genes helps inform strategies to mitigate the impact of antimicrobial resistance on both animal and human health.

The presence of class 1 integrons in all isolates is an important finding that suggests the potential for co-selection and co-localization of resistance genes, which can further enhance the genetic versatility and antibiotic resistance potential of such isolates (32, 33). These isolates may acquire and accumulate multiple resistance genes, making them more adaptable and resistant to antibiotics. The finding also raises concerns about the effectiveness of treatment options, as the presence of class 1 integrons can contribute to the dissemination of multidrug-resistant strains.

PFGE analysis revealed distinct genetic profiles between animal and human CRE isolates, which is perhaps not surprising given their diverse origins. On the other hand, using MLST, all but one of the isolates were identified as belonging to ST410, a sequence type of a high-risk clone that is associated with antibiotic resistance (34). This ST has been frequently linked to CR *E. coli* infections in both humans and dogs globally, including in regions like Thailand (35–37). Furthermore, the ST410 clone exhibits enhanced pathogenic potential, leading to severe or recurrent bloodstream infections in humans, with the capability for interspecies transmission (34). In addition, the previous detection of *E. coli* and *K. pneumoniae* clones shared between owners and their pets (38) supports the potential for transmission of carbapenemase-producing bacteria, and emphasizes the need for intensified surveillance to detect the occurance of CRE under the One Health approach.

The three *K. pneumoniae* isolates from dogs belonged to ST16, ST147, and ST15, suggesting the circulation of varied specific lineages in the canine population. Similarly, the variable ST types identified among human CRK isolates indicate a diverse range of strains contributing to the human burden of carbapenem resistance (39–41). CRE are most likely spread through the horizontal transfer of plasmids carrying carbapenemase genes (42, 43). This horizontal transfer can occur within and between animal and human populations, facilitating the transmission of resistant strains (44, 45).

The findings of this study underscore the importance of comprehensive monitoring and genetic characterization of CRE isolates from companion animals such as dogs and their potential to act as a source of resistance if transferred to human isolates. It is important to note that factors such as environmental contamination or independent acquisition from common sources, also may play a significant role in the spread of carbapenem resistance (46, 47). Understanding the genetic mechanisms and potential routes of transmission of CRE between companion animals and humans is crucial for effective control and prevention strategies. The current findings contribute to the growing body of knowledge regarding the complex dynamics of carbapenem resistance in both animal and human populations, highlighting the need for a One Health approach to address the public health concerns associated with CRE dissemination. Further research is warranted to explore additional factors influencing the transmission dynamics and to develop targeted interventions to mitigate the spread of carbapenem resistance in both human and veterinary healthcare settings.

## 5 Conclusion

This study elucidates the genetic characteristics of CRE isolated from canine and human clinical samples. Our findings indicate that while these resistant bacteria are present in both dogs and humans, there was no direct evidence of clonal transmission between the two hosts. This finding may have been influenced by the relatively small number of isolates examined and the origin of the isolates from canine and human patients with no obvious physical connections. Nevertheless, the available data suggests the existence of independent acquisition and transmission pathways for this resistance. Given the public health implications of CR infections, a comprehensive genomic approach to investigation, such as through complete WGS, will be critical for tracing the potential of mobile genetic transmission and developing appropriate intervention strategies.

## Data Availability

The datasets presented in this study can be found in online repositories. The names of the repository/repositories and accession number(s) can be found in the article/Supplementary material.

## Footnotes

^1^ https://usegalaxy.eu

^2^ http://www.genomicepidemiology.org/
