# Supplementary material for: Genetic characterization and clonal analysis of carbapenemase-producing Escherichia coli and Klebsiella pneumoniae from canine and human origins
Source: Front Vet Sci. 2024 Nov 25;11:1464934. doi: 10.3389/fvets.2024.1464934 (PMC11626800; doi:10.3389/fvets.2024.1464934)
Supplement: Supplementary file 1 [file Image_1.pdf]

(A)

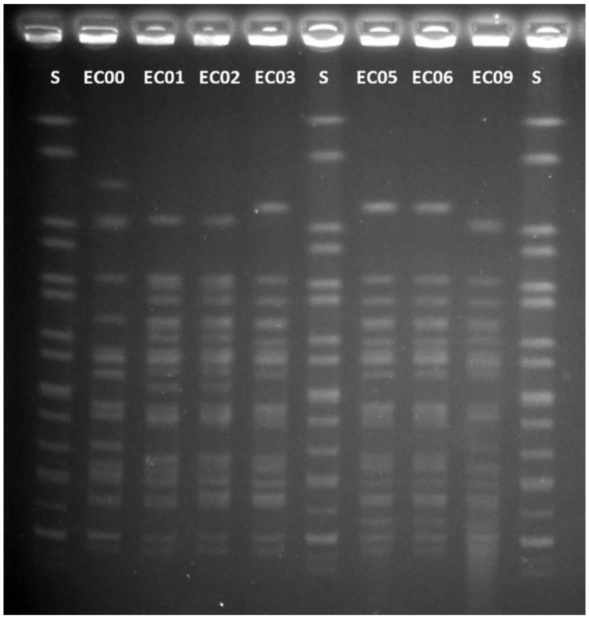

(B)

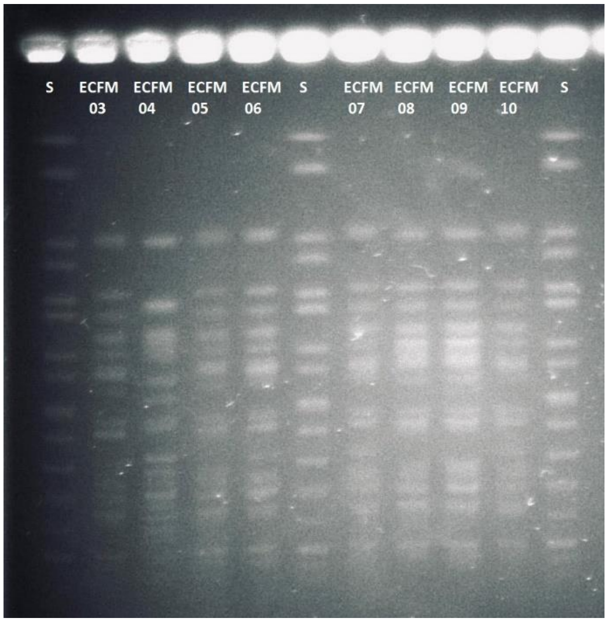

Supplementary figure 1. Pulsed-field gel electrophoresis gel pictures of carbapenem resistant *E. coli* isolates from dogs (A) and humans (B). (S= *Salmonella* as standard)

(A)

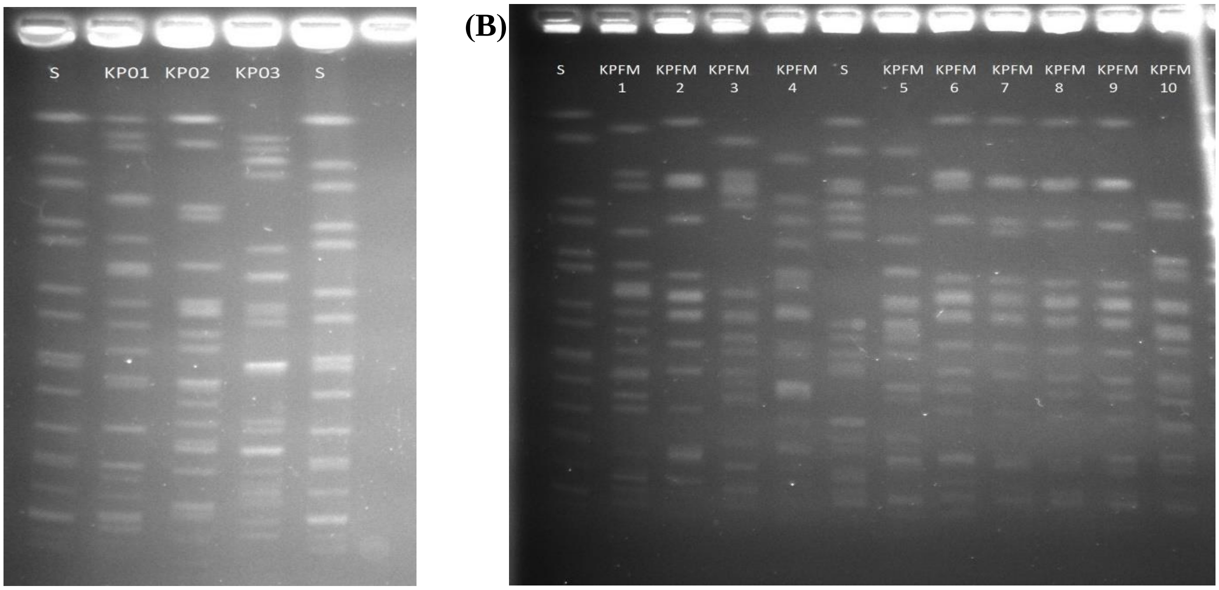

Supplementary figure 2. Pulsed-field gel electrophoresis gel pictures of carbapenem resistant *K. pneumoniae* isolates from dogs (A) and humans (B). (S= Salmonella as standard)
