# Supplementary material for: Genetic characterization and clonal analysis of carbapenemase-producing Escherichia coli and Klebsiella pneumoniae from canine and human origins
Source: Front Vet Sci. 2024 Nov 25;11:1464934. doi: 10.3389/fvets.2024.1464934 (PMC11626800; doi:10.3389/fvets.2024.1464934)
Supplement: Supplementary file 5 [file Table_4.docx]

**Supplementary Table 3:** Matrix of SNP pair counts for carbapenemase-producing *Escherichia coli* in this study (n = 15) compared to reference strain *E. coli* K12 (accession no. NC_000913.2). SNP variant counts 57,359 from 4,007,415 locations, with 86.37% reference coverage.

|  | EC00 | EC01 | EC02 | EC03 | EC05 | EC06 | EC09 | ECFM03 | ECFM04 | ECFM05 | ECFM06 | ECFM07 | ECFM08 | ECFM09 | ECFM10 | reference |
| --- | --- | --- | --- | --- | --- | --- | --- | --- | --- | --- | --- | --- | --- | --- | --- | --- |
| EC00 | 0 | 801 | 801 | 802 | 802 | 803 | 800 | 65 | 47962 | 801 | 64 | 45 | 800 | 45 | 799 | 24324 |
| EC01 | 801 | 0 | 0 | 5 | 5 | 6 | 3 | 824 | 48027 | 32 | 815 | 808 | 31 | 808 | 30 | 24176 |
| EC02 | 801 | 0 | 0 | 5 | 5 | 6 | 3 | 824 | 48027 | 32 | 815 | 808 | 31 | 808 | 30 | 24176 |
| EC03 | 802 | 5 | 5 | 0 | 2 | 5 | 2 | 825 | 48026 | 33 | 814 | 807 | 30 | 809 | 31 | 24175 |
| EC05 | 802 | 5 | 5 | 2 | 0 | 3 | 2 | 825 | 48024 | 33 | 814 | 805 | 28 | 809 | 31 | 24173 |
| EC06 | 803 | 6 | 6 | 5 | 3 | 0 | 3 | 824 | 48027 | 32 | 815 | 808 | 31 | 808 | 30 | 24174 |
| EC09 | 800 | 3 | 3 | 2 | 2 | 3 | 0 | 823 | 48026 | 31 | 814 | 807 | 30 | 807 | 29 | 24175 |
| ECFM03 | 65 | 824 | 824 | 825 | 825 | 824 | 823 | 0 | 47935 | 824 | 53 | 44 | 825 | 42 | 822 | 24295 |
| ECFM04 | 47962 | 48027 | 48027 | 48026 | 48024 | 48027 | 48026 | 47935 | 0 | 48033 | 47946 | 47943 | 48028 | 47941 | 48031 | 42083 |
| ECFM05 | 801 | 32 | 32 | 33 | 33 | 32 | 31 | 824 | 48033 | 0 | 815 | 810 | 11 | 808 | 2 | 24178 |
| ECFM06 | 64 | 815 | 815 | 814 | 814 | 815 | 814 | 53 | 47946 | 815 | 0 | 35 | 814 | 35 | 813 | 24306 |
| ECFM07 | 45 | 808 | 808 | 807 | 805 | 808 | 807 | 44 | 47943 | 810 | 35 | 0 | 805 | 10 | 808 | 24301 |
| ECFM08 | 800 | 31 | 31 | 30 | 28 | 31 | 30 | 825 | 48028 | 11 | 814 | 805 | 0 | 809 | 9 | 24177 |
| ECFM09 | 45 | 808 | 808 | 809 | 809 | 808 | 807 | 42 | 47941 | 808 | 35 | 10 | 809 | 0 | 806 | 24297 |
| ECFM10 | 799 | 30 | 30 | 31 | 31 | 30 | 29 | 822 | 48031 | 2 | 813 | 808 | 9 | 806 | 0 | 24176 |
| reference | 24324 | 24176 | 24176 | 24175 | 24173 | 24174 | 24175 | 24295 | 42083 | 24178 | 24306 | 24301 | 24177 | 24297 | 24176 | 0 |
| min: 0 max: 48033 | | | | | | | | | | | | | | | | |

**Supplementary Table 4:** Matrix of SNP pair counts of carbapenemase-producing *Klebsiella pneumoniae* in this study (*n* = 6) with reference strain *K. pneumoniae*sequence MGH78578 (accession no. NC_009653) (SNP variant count: 48,282 out of 4,600,034 total locations, 80.77% reference coverage).

|  | KP01 | KP02 | KP03 | KPFM 1 | KPFM 3 | KPFM 4 | reference |
| --- | --- | --- | --- | --- | --- | --- | --- |
| KP01 | 0 | 20273 | 20507 | 54 | 20260 | 20700 | 18917 |
| KP02 | 20273 | 0 | 20722 | 20261 | 18513 | 20467 | 17983 |
| KP03 | 20507 | 20722 | 0 | 20493 | 20644 | 21067 | 19244 |
| KPFM 1 | 54 | 20261 | 20493 | 0 | 20248 | 20688 | 18903 |
| KPFM 3 | 20260 | 18513 | 20644 | 20248 | 0 | 20222 | 18165 |
| KPFM 4 | 20700 | 20467 | 21067 | 20688 | 20222 | 0 | 18923 |
| reference | 18917 | 17983 | 19244 | 18903 | 18165 | 18923 | 0 |
